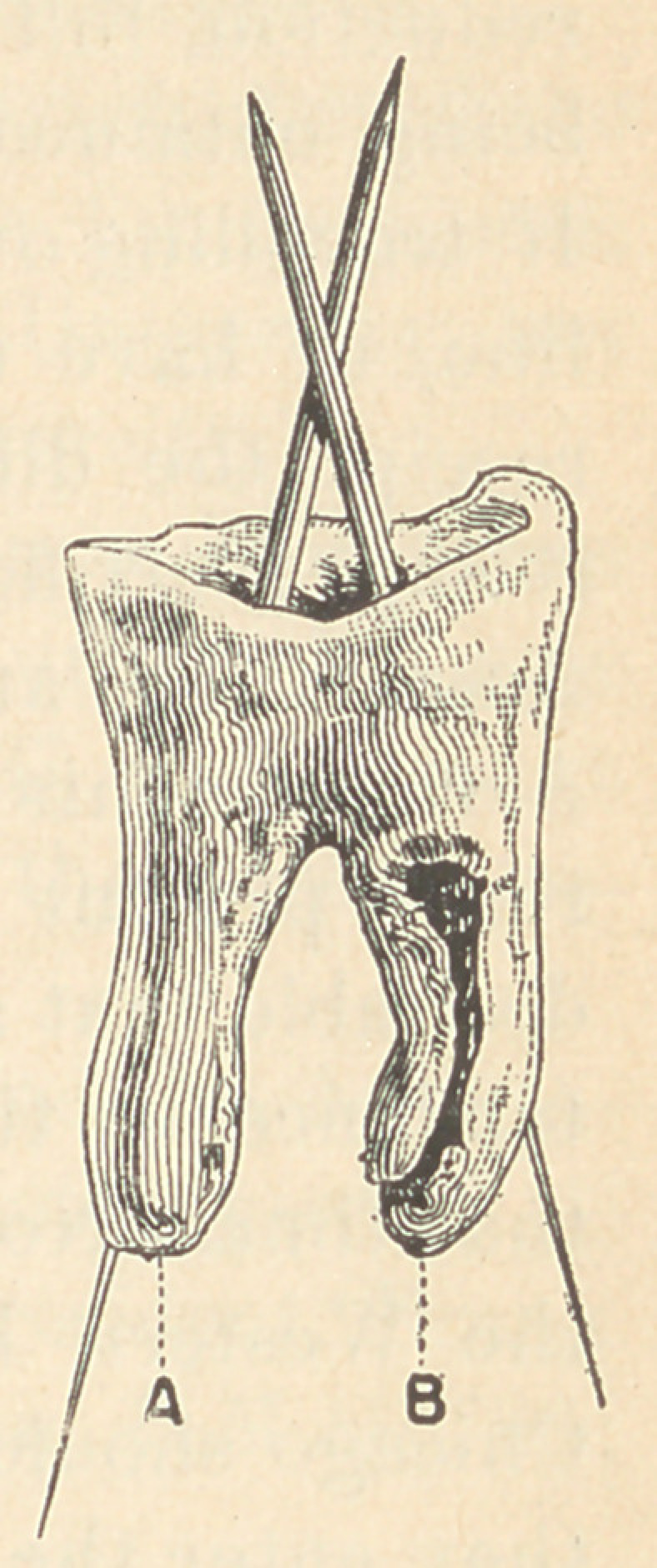# Notes and Comments

**Published:** 1895-08

**Authors:** 


### Notes and Comments.[1]

A Cautionary Signal.—In the April number of the International Dental Journal, under "Selections," was published Dr. J. R. Callahan's method of opening root-canals with sulphuric acid, which has brought forth the following note from Dr. F. A. Roy, New Orleans. He says, in part, "The Callahan method is being taken up by dentists everywhere and of all degrees of competence, so some may use it as an easy way to avoid careful work in tedious

[1] The assistant editor solicits contributions for this department,—new methods, new remedies and formulas, or any short practical note which may prove of value to the practitioner or student. Address 1718 Walnut Street, Philadelphia.

operations, despite the doctor's conservative statements. The cut shows a possibility even with careful manipulation, a certainty with careless work. This tooth was selected because the root ends appeared thickened and dense, with small canals. After persistent effort the broach found its way through the anterior root *via* the buccal canal, but not through the foramen B. The root was cut open to expose the lingual canal, which shows plainly that probably no broach, with or without acid, and certainly no drill, could be made to follow the canal.

"Then the posterior root was cut or drilled. $H_2SO_4$ cuts and really enables one to drill with a broach, and, as with any drill unless carefully handled, with possible action understood, we may be misled. See the result in this nearly straight root. The foramen is at A. The result in the anterior root is somewhat discouraging. Drilling with acid is not so much easier nor more sure in following canals than older methods. Not all crooked or obstructed canals may be opened in a few minutes, and the careless may manufacture canals in the alveolus."

---

Simple Method of cleaning Impression Trays.—"Give the impression trays a coating of sweet oil with a woollen cloth dipped in the oil. Put them in strong soap-suds (made with soap shavings or powder), boil, and wipe dry. Now polish with whiting by using a soft woollen cloth or fine leather. In this way you can keep your trays bright and clean, and the plaster will not adhere to them."—E. B. Edgers, D.D.S.

## Figures and Tables

**Figure f1:**